# Evaluation of adenoviral vector Ad19a encoding RSV-F as novel vaccine against respiratory syncytial virus

**DOI:** 10.1038/s41541-024-01001-z

**Published:** 2024-10-29

**Authors:** Jana Fuchs, Julian Hübner, Anna Schmidt, Pascal Irrgang, Clara Maier, Ana Vieira Antão, Friederike Oltmanns, Christian Thirion, Dennis Lapuente, Matthias Tenbusch

**Affiliations:** 1 Institute of Clinical and Molecular Virology, University Hospital Erlangen, Friedrich-Alexander-Universität (FAU) Erlangen-Nürnberg, Schlossgarten 4, 91054 Erlangen, Germany; 2 Sirion Biotech, Am Klopferspitz 19, 82152 Martinsried, Germany; 3 FAU Profile Center Immunomedicine (FAU I-MED), Friedrich-Alexander-Universität (FAU) Erlangen-Nürnberg, Schlossplatz 1, D-91054 Erlangen, Germany

**Keywords:** Viral infection, Live attenuated vaccines, Vaccines

## Abstract

Respiratory syncytial virus (RSV) is the leading cause of severe lower respiratory tract infections in infants and toddlers. Since natural infections do not induce persistent immunity, there is the need of vaccines providing long-term protection. Here, we evaluated a new adenoviral vector (rAd) vaccine based on the rare serotype rAd19a and compared the immunogenicity and efficacy to the highly immunogenic rAd5. Given as an intranasal boost in DNA primed mice, both vectors encoding the F protein provided efficient protection against a subsequent RSV infection. However, intramuscular immunization with rAd19a vectors provoked vaccine-enhanced disease after RSV infection compared to non-vaccinated animals. While mucosal IgA antibodies and tissue-resident memory T-cells in intranasally vaccinated mice rapidly control RSV replication, a strong anamnestic systemic T-cell response in absence of local immunity might be the reason for immune-mediated enhanced disease. Our study highlighted the potential benefits of developing effective mucosal against respiratory pathogens.

## Introduction

The human respiratory syncytial virus (RSV) is one of the main causes of severe lower respiratory tract infections in young infants and elderly. Nearly all children experience an infection during the RSV seasons within their first 2 years^1,2^. In the course of a primary infection, 15–50% show an involvement of the lower airways from which in turn 1–3% require hospitalization^3^. Worldwide, it was estimated that annually about 30 million of RSV-induced episodes of acute lower respiratory tract infection occurred leading, to about 60,000 deaths in children under the age of five^4^. Additionally, long-term consequences, such as airway hypersensitivity and asthma, are more likely to be developed after a severe RSV infection^5–7^. Generally, elderly and immunocompromised individuals are at higher risk of severe disease after RSV infection^8,9^. Since natural infections do not induce persistent immunity^10,11^, a vaccine that provides efficient and long-lasting protection against RSV is of utmost importance. Until 2023, no prophylactic vaccine was licensed and the only preventive measure was a passive antibody treatment for high-risk children in form of a monoclonal antibody (Palivizumab) targeting the F-Protein of RSV^12–14^. A newly licensed antibody (Nirsevimab) has an increased half-life and avidity, which renders a single dose during the RSV season sufficient for efficient protection^15–18^. In many countries, this lead to the recommendation to apply it to all children during their first RSV season. Most recently, with Arexvy (GlaxoSmithKline) and ABRYSVO^TM^ (Pfizer), two vaccines have been approved by the FDA for the prevention of RSV in older adults. Both vaccines are based on the stabilized prefusion RSV-F protein and demonstrated efficacy against severe lower respiratory tract infection in clinical phase III trials^19,20^. ABRYSVO^TM^ (Pfizer) has been also approved to be used in pregnant women as maternal vaccination^21,22^.

For a long time, the development of new vaccines was impeded by the observation of vaccine-enhance respiratory disease (ERD) in an early clinical trial with a formaline-inactivated (FI-) RSV vaccine. In this study, an increased hospitalization rate and enhanced disease severity was observed after community-acquired RSV infections in children vaccinated with FI-RSV compared to the placebo group^23^. Several immune mechanisms have been postulated to induce ERD in animal models of RSV infection. Some studies reported on vaccine-induced antibodies with low avidity for protective epitopes as the most probable reason for vaccine failure^24–26^, whereas others found distinct CD4^+^ T-cell subsets to be responsible for specific disease parameters found in ERD patients. A T helper (Th)2-biased immune response resulted in airway hyperreactivity and mucus hypersecretion, whereas the Th1-associated cytokine TNFα could be associated with airway obstruction and weight reduction^27^.

In contrast, efficient protection against RSV infection correlates with local cellular immunity in the lungs, such as IFNγ producing CD4^+^ T-cells, which have been identified as a protective measure against infection and subsequent lung inflammation^28^. Furthermore, FI-RSV immunization failed to induce RSV-specific memory CD8^+^ T-cells^29,30^, which have been reported to be critical for optimal protection^30–32^. Next to their direct antiviral effector functions, RSV-specific CD8^+^ T-cells reduce the number of Th2 cells in the lung^29,33^ and thus reduce Th2-related inflammatory processes^28,33^. Specifically, RSV-specific lung resident memory T-cells (T_RM_) have been correlated to protection against RSV-mediated disease^34–37^.

However, except one live-attenuated flu vaccine, all licensed vaccines against respiratory viruses are applied intramuscularly and are therefore conceptualized to induce protective systemic immune responses, most probably in form of neutralizing antibodies. However, the large number of SARS-CoV-2 breakthrough infections in vaccinated individuals might suggest that the low level of mucosal immune responses after systemic vaccination will provide sterile immunity and still allows transmission of SARS-CoV-2^38–40^. Although not formally shown yet, this might be also a consequence for other respiratory pathogens.Therefore, the establishment of new vaccine strategies focusing on mucosal application are thought to be a key to reduce the global health burden by respiratory viruses, such as RSV, influenza A Virus (IAV) or SARS-CoV-2^37,40–46^. Several pre-clinical studies reported on vaccine-induced mucosal immunity capable to protect against subsequent RSV infections in animal models or human challenge studies^37,44,47–49^. Our group has also demonstrated that a systemic prime immunization with DNA or RNA-based vaccines followed by mucosal applications of adenoviral vector vaccines induced balanced systemic and local immune responses, which efficiently protects against RSV, IAV or SARS-CoV-2^37,42,50^.

Due to potential limitations and concerns to the use of Ad5-based vaccines^51–54^, we evaluated a rare serotype adenoviral vector vaccine (rAd19a) encoding the RSV F protein as booster modality after a systemic DNA prime immunization. We could demonstrate that a mucosal but not a systemic rAd19a boost induces T_RM_ and mucosal immunoglobulin A (IgA) in the respiratory tract. These mucosal responses likely contribute to the efficient control of virus replication and prevention of disease progression upon RSV infection. Moreover, mucosal immunity prevented vaccine-enhanced RSV disease that is otherwise observed upon an intramuscular immunization with rAd19a.

## Results

### Humoral and cellular responses induced by mucosal and systemic rAd19a boost vaccination

**Fig. 1:**
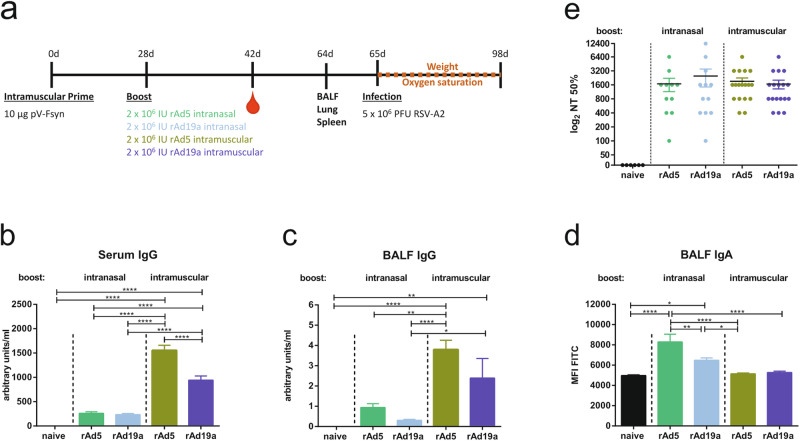
Humoral immune response after rAd5 and rAd19a boost. **a** BALB/c mice were primed intramuscularly with an F-encoding DNA plasmid (10 µg plasmid) followed by electroporation and boosted 28 days later either intramuscularly (i.m.) or intranasally (i.n.) with rAd5 or rAd19a viral vectors encoding for F (2 ×10^6^ infectious units per vector). Serum antibody responses were analysed 14 days and mucosal immune responses 36 days after boost immunization. F-specific IgG (**b** and **c**) and IgA (**d**) were examined by a flow cytometric assay using a 293A cell line stably expressing F. For IgG arbitrary units were calculated according to standard mouse serum and mean fluorescence intensities (MFI) were shown for IgA. The MFI of naïve samples indicate the background values of the assay. Bars represent mean values with +SEM (Serum: rAd5 (i.n.) *n* = 11, rAd19 (i.n.) *n* = 12, other groups *n* = 18; BALF: rAd5 (i.n.) *n* = 11, other groups *n* = 12). **e** Neutralization antibodies against RSV in sera were analysed by microneutralization assay 14 days after boost immunization. Depicted are the individual PRNT_50_ values with the group´s mean values with ±SEM. (naive *n* = 6, rAd5 (i.n.) *n* = 11, rAd19 (i.n.) *n* = 12, other groups *n* = 18). Data were analysed by one-way ANOVA followed by Tukey´s multiple comparison test. Statistically significant differences were indicated among all groups (*p < 0.05; **p < 0.005; ****p < 0.0001).

To assess the immunogenicity of our alternative adenoviral vector, mice primed with a DNA vaccine encoding codon-optimized full-length RSV-F were boosted after four weeks with rAd19a-F either via the intranasal or intramuscular route (Fig. 1a). The established vector, based on serotype 5, referred to as rAd5-F, was tested in direct comparison as benchmark control. Both replication-deficient vectors encode for the non-stabilized wild-type RSV-F protein and led to similar expression levels after transduction of eukaryotic cells, such as A549. Furthermore, the presence of the pre-fusion conformation on the plasma membrane of transduced cells was confirmed by preF-specific antibodies and flow cytometry (Supplementary Fig. 1).

A flow cytometer-based technique was also employed to analyse vaccine-induced RSV-F-specific antibody responses in serum and BALF. The intramuscular boost immunization with both adenoviral vectors resulted in robust F-specific antibody responses with significantly higher IgG levels in mice boosted with rAd5 compared to rAd19a. Intranasally applied, both vectors induced comparable RSV-F-specific serum IgG levels, but significantly less compared to the intramuscular immunization (Fig. 1b). The IgG levels detected in the BALF mirrored the serum IgG levels, but in sharp contrast mucosal IgA antibodies were only detectable in animals having received an intranasal boost immunization (Fig. 1c, d). Again, the rAd5 was significantly more potent than the rAd19a vector. Furthermore, the capability of the vaccine-induced antibodies to bind the postfusion or prefusion conformation was analysed by ELISA using either postF or preF protein as coating agent (Supplementary Fig. 2). Here, in all vaccinated groups antibodies reactive to both proteins could be detected with the above described hierarchy. The intramuscular immunization with rAd5-F had significant higher IgG serum level to preF and postF than all other groups. Importantly, the ratio of postF/preF antibodies were not altered between the two vector platforms. The same was true for postF- and preF-specific IgA responses in the BALF, which were only detectable in the two intranasally boosted animals (Supplementary Fig. 2).As neutralizing antibodies are reported to be a correlate of protection^55^, neutralizing activity against RSV-A2 was evaluated in serum on day 42. Although the NT50 values showed some degree of variation, all immunization strategies induced antibodies with comparable neutralization capacity. No neutralizing activity was seen in naive animals (Fig. 1e).

**Fig. 2:**
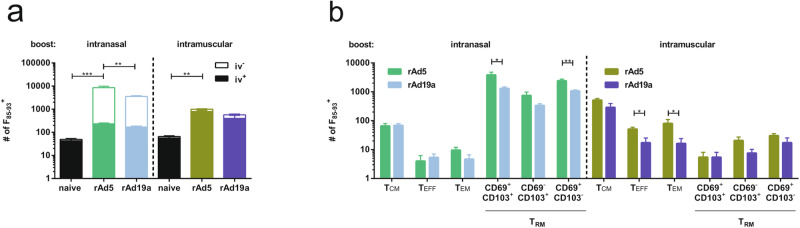
CD8^+^ T-cell subpopulations in the lung. Balb/c mice were immunized as described before and lymphocytes from lungs were examined 36 days post boost. Antigen experienced CD8^+^ T-cells were identified by F_85-93_-specific pentamer staining and intravascular (iv) CD45 pan staining. CD45-labelled cells (iv^+^) were defined as circulating and iv protected (iv^-^) as tissue resident memory cells. **a** The absolute number of F_85-93_^+^ CD8^+^ with the relative contribution of iv^+^ and iv^-^ cells was summarized for each group. Statistical analysis was performed over whole population. **b** Within the iv^+^ F_85-93_^+^ CD8^+^ population central memory T-cells (T_CM_; CD127^+^KLRG1^-^CD69^-^CD103^-^), effector T-cells (T_EFF_; CD127^-^KLRG1^+^) and effector memory T-cells (T_EM_; CD127^+^KLRG1^+^) were determined, whereas within the iv^-^ population tissue resident memory T-cells (T_RM_; KLRG1^-^CD103^+^CD69^+^ or KLRG1^-^CD103^+^CD69^-^ or KLRG1^-^CD103^-^CD69^+^) were defined^100^. Bars represent mean values with +SEM; naive *n* = 3, rAd5 (i.n.) *n* = 5, other groups *n* = 6. Data were analysed by one-way ANOVA followed by Tukey´s multiple comparison test. Statistically significant differences were indicated only among the used vaccine vectors within the intranasal resp. intramuscular group (*p < 0.05; **p < 0.005).

Five weeks after the boost immunization, systemic and local cellular immune responses were evaluated in the spleen and in the lung, respectively. Intravascular staining (iv) with anti-CD45 antibodies was performed to distinguish between circulating and tissue-resident T-cells in the lung. F-specific T-cells were identified by pentamer staining. In all vaccinated animals, T-cells specific for the immunodominant F_85-93_ peptide could be readily detected. In absolute numbers, unlabelled (iv^-^) F-specific T_RM_ were most abundant after the intranasal booster immunizations, whereas the intramuscular application induced circulating T-cells (iv^+^) more efficiently. Analysing the memory phenotype in more detail, CD69^+^CD103^+^ and CD69^+^CD103^-^ T_RM_ were the dominating subpopulations in mucosally immunized mice, with rAd5 being more efficient in inducing these T-cell populations (rAd5: 3835.2 ± 974.8 and rAd19a: 1312.0 ± 176.2 CD69^+^CD103^+^). In contrast, the intramuscular prime-boost schedules mainly led to circulating memory phenotypes dominated by central memory T-cells (T_CM_; Fig. 2b).

**Fig. 3:**
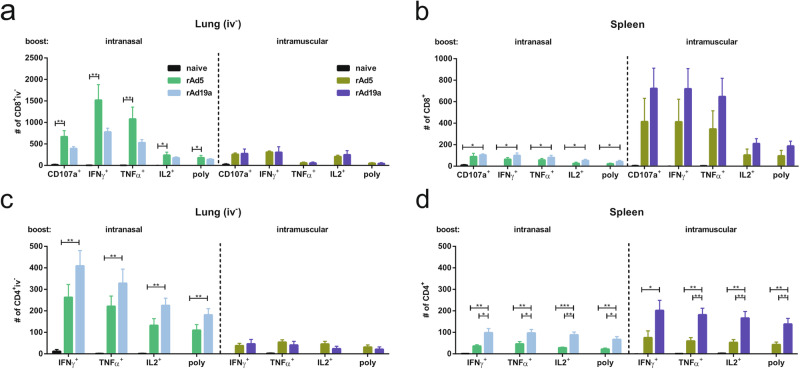
F-specific T-cell responses after immunization in lung and spleen. Balb/c mice were immunized as described before and lymphocytes from lungs and spleens were restimulated with peptide pools covering immunogenic parts of F 36 days post boost. CD8^+^ (**a** and **b**) and CD4^+^ (**c** and **d**) T-cell responses were analysed by staining for the degranulation marker CD107a and intracellular staining for inflammatory cytokines IFNγ, TNFα and IL2. **a**, **c** Lung responses of tissue resident (iv^-^) T-cells were analysed. Total numbers of different populations among CD8^+^ and CD4^+^ T-cells are shown. Bars represent mean values with +SEM; naive *n* = 3, rAd5 (i.n.) *n* = 5, other groups *n* = 6. Data were analysed by one-way ANOVA followed by Tukey´s multiple comparison test. Statistically significant differences were indicated only among the used vaccine vectors within the intranasal resp. intramuscular group (*p < 0.05; **p < 0.005). poly: polyfunctional T-cell populations positive for all assessed markers.

T-cell functionality was analysed by re-stimulation of isolated lymphocytes with immunodominant peptides from the F protein followed by intracellular cytokine staining. In line with the pentamer data, the intranasal booster immunization led to high levels of tissue-resident (iv^-^), cytokine-secreting CD8^+^ T-cells in the lung (Fig. 3a), while the intramuscular booster mainly induced systemic T-cell responses in the spleen (Fig. 3b). Interestingly, the rAd5 vector was more efficient in inducing CD8^+^ T_RM_ in the lung, whereas rAd19a more efficiently established T-cell memory present in the spleen (Fig. 3a, b).

We observed a similar compartmentalization of the CD4^+^ T-cell responses after intranasal and intramuscular adenoviral booster immunizations (Fig. 3c). However, in contrast to the CD8^+^ T-cell responses, absolute numbers of F-specific CD4^+^ T-cells were overall higher after rAd19a immunization compared to vaccination with rAd5. While this difference was not statistically significant in the T_RM_ compartment after the intranasal immunizations, systemic F-specific CD4^+^ T-cell responses in the spleen were significantly higher in the rAd19a treated animals, independent of the route of administration (Fig. 3d). Thus, rAd19a and rAd5 show specific immunogenicity profiles that differ among T-cell subsets.

### A mucosal rAd19a booster vaccination efficiently protects against RSV disease, while ERD is observed after systemic booster immunization

**Fig. 4:**
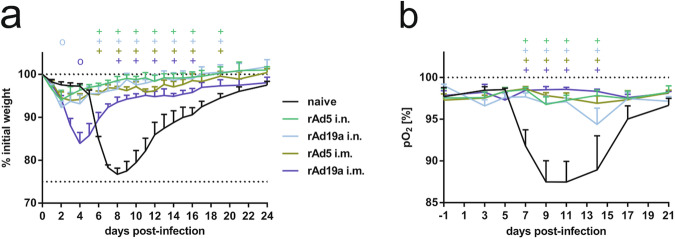
Protective efficacy against RSV infection. Balb/c mice were immunized as described before. **a**, **b** 37 days after boost immunization, mice were challenged with 5 ×10^6^ PFU RSV-A. All animals were monitored daily for body weight (**a**) and every second day for oxygen saturation (**b**). Time points show group´s mean values with +SEM; all groups *n* = 6. Data were analysed by two-way ANOVA followed by Dunnett´s multiple comparison test. Statistically significant differences were indicated among naive and vaccinated groups; (o: statistically significant worse than naive; +: statistically significant better than naive).

To evaluate the vaccine efficacy, immunized mice were infected with 5 ×10^6^ PFU RSV-A2 on day 65. In naive animals, weight loss was observed as early as day five post-infection reaching peak weight loss of about 25% at day eight. Afterwards, all animals regained weight and fully recovered until day 24 post-infection (Fig. 4a). In contrast, both rAd5 vector-boosted groups and the intranasal rAd19a boosted cohort were almost completely protected from disease manifestation, except for a minimal weight loss during the first three days of infection, which even reached statistical significance for the rAd19 group at day 2 However, animals that received an intramuscular boost with rAd19a showed a pronounced weight loss peaking at day four (83.9% ±2.6%), before they recovered significantly faster than unvaccinated animals. Particularly the very early and pronounced weight loss in this group was unexpected. As an additional parameter for disease progression, the oxygen saturation was monitored throughout the infection by pulse-oximetry^56^. In naive mice, the drop in oxygen saturation mirrored the weight curve and lowest oxygen levels were recorded with 87.5% ±2.55% on day nine post-infection. It took about three weeks to return to normal levels (Fig. 4b). The oxygen levels in all immunized animals remained in a physiological range throughout the observation period, even in the animals of the rAd19a i.m. group which showed substantial weight loss. Therefore, our prime-boost immunizations were at least partially protective against subsequent RSV infections. However, the DNA prime followed by a systemic rAd19a boost scheme induced an enhanced disease phenotype early after infection, which might be considered as a form of ERD. To address potential differences in the quality of the anti-F antibody response induced by the two vector platforms as reason for the differential outcome after the intramuscular application, we quantified anti-RSV-F IgG1 and IgG2 antibodies in serum before and after the challenge. As seen before, Ad5-F induced higher levels of F-specific IgG antibodies after intramuscular application than Ad19a-F, which was in case of the IgG2a subclass statistically significant (Supplementary Fig. 3). In contrast, the levels of F-specific IgG1 and IgG2 were the highest after the challenge in the rAd19a-F i.m. group. The increase of both IgG subclasses was significantly higher than in the other vaccine groups, which might reflect higher antigen exposure during the RSV infection (Supplementary Fig. 3).

### Efficient mucosal immunity protects against immune-mediated ERD

**Fig. 5:**
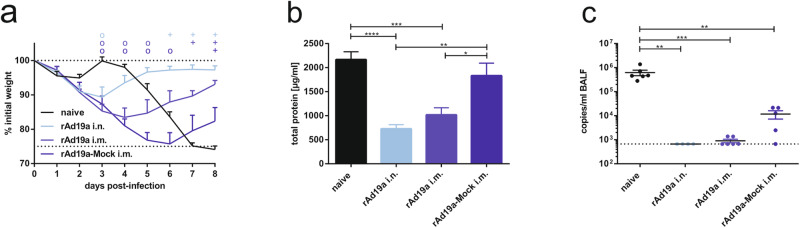
Influence of rAd19a boost immunization on protective efficacy against RSV. BALB/c mice were primed intramuscularly with an F-encoding DNA plasmid (10 µg plasmid) followed by electroporation and boosted 28 days later intranasally (i.n.) or intramuscularly (i.m.) with rAd19a viral vector encoding for F or the influenza nucleoprotein (NP; mock) (2 ×10^6^ infectious units per vector). 37 days after second immunization, all mice were challenged with 5 ×10^6^ PFU RSV-A. **a** Animals were monitored daily for body weight. Time points show group´s mean values with +SEM; rAd19a-Mock (i.m.) *n* = 5, other groups *n* = 6. Data were analysed by Friedman test followed by Dunn´s multiple comparison test. Statistically significant differences were indicated among naive and vaccinated groups; (o: statistically significant worse than naive; +: statistically significant better than naive). **b** Tissue damage was measured indirectly by protein content in the BALF eight days after infection. Bars represent mean values with +SEM; rAd19a-Mock (i.m.) *n* = 5, other groups *n* = 6. Data were analysed by one-way ANOVA followed by Tukey´s multiple comparison test. Statistically significant differences were indicated among all groups (*p < 0.05; **p < 0.005; ***p < 0.0005; ****p < 0.0001). **c** At day eight, viral loads in BALF samples were measured by qRT-PCR. Depicted are the individual copies/ml BALF with the group´s mean values with ±SEM; rAd19a (i.n.) *n* = 4, rAd19a-Mock (i.m.) *n* = 5, other groups *n* = 6. The qRT-PCR´s detection limit was 667 copies/ml BALF and is marked with a dotted line. Data were analysed by one-way ANOVA followed by Tukey´s multiple comparison test. Statistically significant differences were indicated among all groups. (**p < 0.005; ***p < 0.0005).

In a first attempt to understand the origin of this enhanced disease phenotype, we compared different rAd19a boost modalities in mice. Specifically, mice were initially immunized with RSV-F-encoding DNA and boosted with rAd19a-F intranasally or intramuscularly as before. An additional group receiving a rAd19a-NP vector intramuscularly was included as mock control. The latter should indicate whether the DNA-induced primary response leads to similar pathologies. Five weeks after the second vaccination, the immunized animals and a naive control group were challenged with 5 ×10^6^ PFU RSV-A2. The impact on viral replication was analysed on day eight. Monitoring of the weight confirmed the early and profound weight loss in animals boosted intramuscularly with rAd19a-F and the rapid recovery after intranasal booster immunization (Fig. 5a). However, the group of mice that had received the rAd19a-NP vector showed almost comparable weight loss to naive animals, but with a completely different kinetic. The mock group showed a constant loss of weight from day one until day five and six, whereas naive animals started to lose weight at day fore, which was in line with the previous experiment (Fig. 5a).

The integrity of the lung and the epithelial barrier function was indirectly assessed by analysing the protein content in the BALF. In line with the observed weight loss, protein levels were low in both groups having received the rAd19a-F vector (i.m. or i.n.) and significantly higher in the naive and rAd19a-Mock boosted animals (Fig. 5b). However, rAd19a-Mock treated animals had still significantly lower viral loads than the non-immunized animals as quantified by qRT-PCR, which indicates that the DNA-induced primary response provides partial protection (Fig. 5c). In the other two groups, the RNA copy numbers were below the detection limit in all except two animals of the rAd19-F i.m. group (Fig. 5c). To exclude intrinsic properties of the new rAd19a vector system as cause of the increased immunopathology, we also applied an intramuscular rAd5-NP booster immunization in DNA primed mice. This revealed highly comparable results in regard to weight loss, tissue damage and viral loads as rAd19a-NP (Supplementary Fig. 4).

Therefore, our results suggest that systemic immunity induced by vaccination can provide protection against viral replication, but at a low level and in absence of efficient local immunity it might also lead to immune-mediated pathologies.

**Fig. 6:**
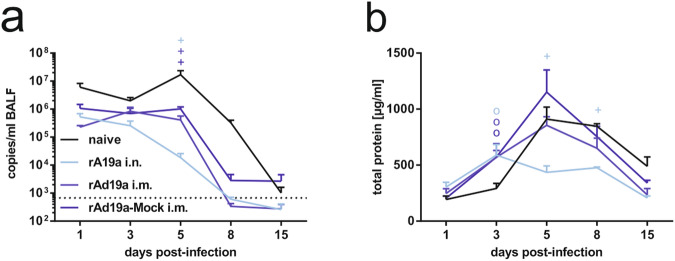
Course of disease in dependency of prior vaccination during early RSV infection. BALB/c mice were primed intramuscularly with an F-encoding DNA plasmid (10 µg plasmid) followed by electroporation and boosted 28 days later either intranasally (i.n.) or intramuscularly (i.m.) with rAd19a viral vector encoding for F or the influenza NP (mock) (2 ×10^6^ infectious units per vector). 38 days after second immunization, all mice were challenged with 5 ×10^6^ PFU RSV-A. The experiment started with 25 animals per group. On the time points one, three, five, eight, and 15 days post-infection 5 mice each group were analysed. **a** Viral loads in BALF samples were measured by qRT-PCR. Time points represent mean values with +SEM; all groups per time point *n* = 5. The qRT-PCR´s detection limit was 667 copies/ml BALF and is marked with a dotted line. **b** Tissue damage was indirectly measured by protein content in the BALF after infection. Time points represent mean values with +SEM; all groups per time point *n* = 5. Data were analysed by two-way ANOVA followed by Dunnett´s multiple comparison test. Statistically significant differences were indicated among naive and vaccinated groups; (o: statistically significant worse than naive; +: statistically significant better than naive).

**Fig. 7:**
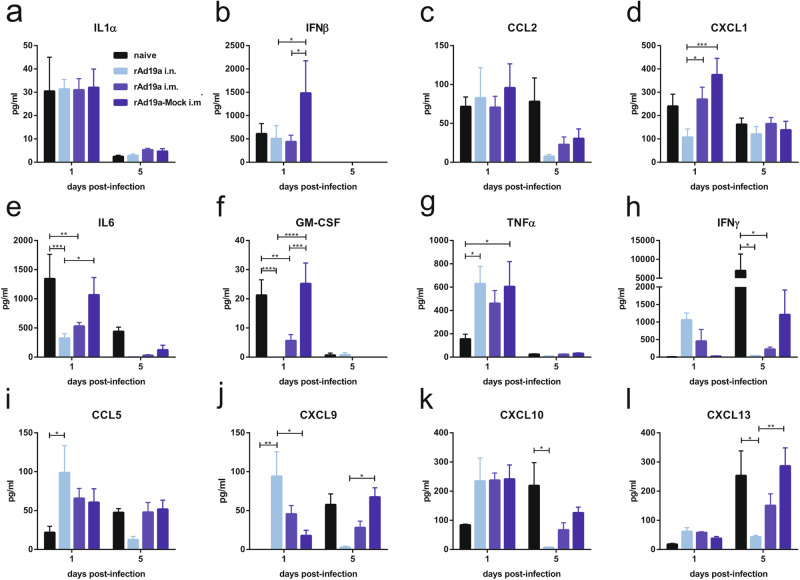
Early inflammation state post RSV infection. Balb/c mice were immunized and challenged as described in Fig. 6. Samples were harvested one and five days post-infection. Levels of IL1α (**a**), IFNβ (**b**), CCL2 (**c**), CXCL1 (**d**), IL6 (**e**), GM-CSF (**f**), TNFα (**g**), IFNγ (**h**), CCL5 (**i**), CXCL9 (**j**), CXCL10 (**k**) and CXCL13 (**l**) were examined in BALF by a Legendplex assay (flow cytometric assay) to get an overview of the early inflammatory state after RSV infection. Bars represent mean values with +SEM; all groups *n* = 5. Data were analysed by one-way ANOVA followed by Tukey´s multiple comparison test. Statistically significant differences were indicated among all groups (*p < 0.05; **p < 0.005).

To shed more light on the underlying immunological processes, we performed a comprehensive kinetic study analysing viral loads, lung tissue damage, inflammatory cytokines, T-cell and antibody responses within the affected lung tissue at several time points after the RSV challenge. Viral load measurements indicated slightly reduced viral RNA copy numbers in all vaccinated groups already at day one compared to the naive group (Fig. 6a). Interestingly, in the following days, viral loads steadily declined in the intranasally boosted animals, whereas in the two other immunized groups viral loads remained at a nearly constant level until day five, which also marked the peak viral load in naive animals. Nevertheless, the viral loads at day five were significantly lower in all vaccine groups confirming the vaccine-mediated control of viral replication in the lung. At day 15, all animals cleared the viral infection except two animals each of the naive and the rAd19-Mock groups (Fig. 6a). Interestingly, the protein levels in the BALF as indicator of lung damage were higher in the immunized animals at day three after the challenge (Fig. 6b). In line with the viral loads, tissue damage further increased and reached its maximum at day five in all groups except the intranasally boosted ones. The latter had significantly lower protein levels in the BALF than the naive animals on day five and eight. The highest degree of tissue damage was observed in the rAd19a-Mock group, which correlates with the highest weight loss at this time point (Fig. 5a). At the end of the observation period, the protein levels were still elevated in the infected control group (naive), potentially reflecting the longer period of viral replication. Using cytometric bead assays, we analysed in total 26 cytokines and chemokines in the BALF samples at each time point to monitor the on-going inflammation (Fig. 7). Some markers of early virus-induced inflammation were not altered and we detected similar levels of IL1α, IFNβ or CCL2 in all mice one day after virus infection as a potential result of pattern-recognition receptor activation in infected cells. (Fig. 7a–c). On the other hand, other inflammatory cytokines at that time point, such as CXCL1, IL6 and GM-CSF, were significantly higher in the animals with more severe disease progression, which might reflect differences in viral replication (naive or rAd19a-Mock) (Fig. 7d–f). Finally, there was a group of cytokines and chemokines: TNF, IFNγ, CCL5, CXCL9, CXCL10 and CXCL13, which were early after infection elevated in the vaccinated but not detectable in the naive animals, suggesting an adaptive immune response as the trigger. Specifically, animals that received the intranasal immunization with rAd19a-F had high levels of CCL5, TNF and IFNγ at day one, potentially indicating a rapid response by local F-specific T_RM_. Since CXCL9 expression is strongly induced by IFNγ, CXCL9 levels were also significantly higher in the protected animals compared to the naive ones at day one (Fig. 7g–l). Interestingly, the expression levels at day five of IFNγ, CCL5, CXCL9, CXCL10 and CXCL13 mirrored the observed trend of tissue damage in the different groups, with the lowest cytokine/chemokine levels in the intranasally boosted animals. Of note, we compare a primary immune response in previously naïve animals partially with anamnestic responses in vaccinated animals. Therefore, the major cytokine-producing cell populations might differ between the different groups, e.g. IFNγ could be either produced by activated NK cells or reactivated memory T-cells. However, the distinct cytokine/chemokine profiles clearly depended on the vaccination status of the mice.

**Fig. 8:**
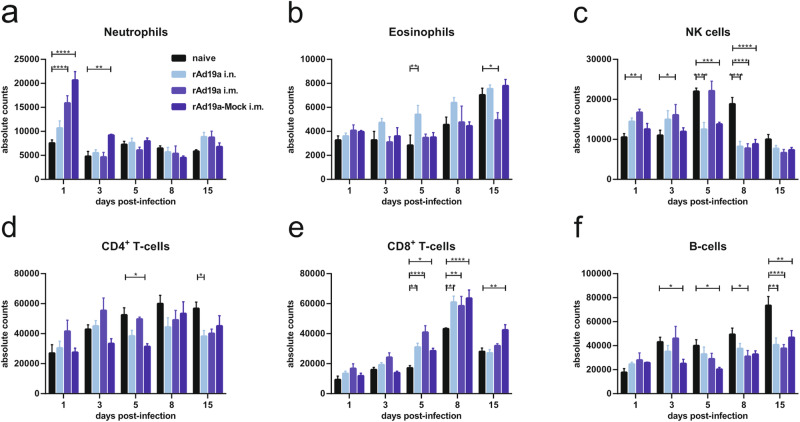
Cell infiltration post RSV infection. Balb/c mice were immunized and challenged as described in Fig. 6. Samples were harvested on respective days post-infection. Lungs were analysed and total numbers of neutrophils (**a**), eosinophils (**b**), NK cells (**c**), CD4^+^ T-cells (**d**), CD8^+^ T-cells (**e**), and B-cells (**f**) are shown (gating seen in Supplementary Fig. 6). Bars represent mean values with +SEM; all groups *n* = 5. Data were analysed by one-way ANOVA followed by Tukey´s multiple comparison test. Statistically significant differences were indicated among all groups (*p < 0.05; **p < 0.005; ***p < 0.0005; ****p < 0.0001).

Next, the cellular infiltrates of the infected lung tissue was analysed by flow cytometry at the indicated time points. At day one post-infection, the number of neutrophils was higher in the vaccinated animals compared to the non-immunized mice reaching statistical significance in both intramuscular boosted groups (Fig. 8a). At all later time points, no differences were observed between the cohorts. Similarly, the level of eosinophils was quite constant within this observation period and there was no sign of severe eosinophilia, which had been discussed in earlier reports on ERD (Fig. 8b). In non-immunized mice, there was an infiltration of NK cells within the first five days after the RSV infection, whereas in vaccinated animals the number of NK cells was slightly elevated at day one and then declined over time (Fig. 8c). In the compartment of adaptive lymphocytes, there was only minimal fluctuation in the absolute number of CD4^+^ T-cells over time (Fig. 8d). In contrast, an infiltration or expansion of CD8^+^ T-cells was detectable in all groups with similar kinetics. The highest levels of CD8^+^ T-cells were measured at day eight post-infection and all vaccinated groups had significantly higher CD8^+^ T-cell counts at day five and eight compared to the non-immunized animals (Fig. 8e). In contrast, the increase of B cells was significantly higher in the lungs of the infected control animals, with a four-fold elevation by day 15. All other groups exhibited only a minor rise (Fig. 8f).

**Fig. 9:**
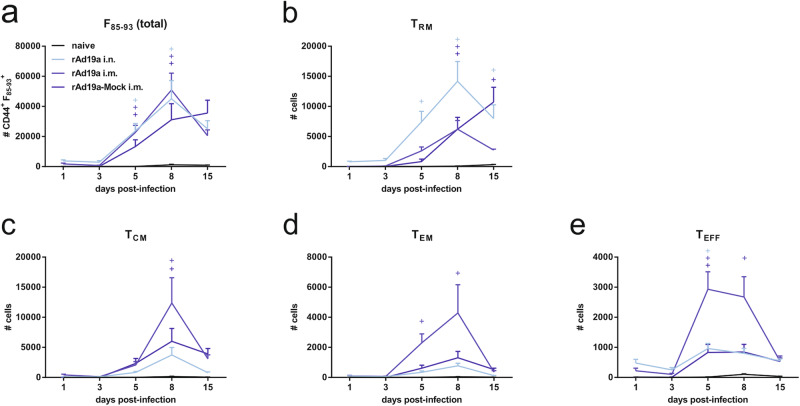
CD8^+^ T-cell subpopulations in the lung during RSV infection in dependency of prior vaccination. Balb/c mice were immunized and challenged as described in Fig. 6 and lymphocytes from lungs were examined on respective days post-infection. Antigen experienced CD8^+^ T-cells were identified by CD44^+^ and F_85-93_-specific pentamer staining. **a** The absolute number of CD44^+^ F_85-93_^+^ CD8^+^ T-cells was summarized for each group. Within the CD44^+^ F_85-93_^+^ CD8^+^ T-cell population tissue resident memory T-cells (T_RM_; KLRG1^-^CD103^+^CD69^+^) (**b**), central memory T-cells (T_CM_; CD127^+^KLRG1^-^CD69^-^CD103^-^) (**c**), effector memory T-cells (T_EM_; CD127^+^KLRG1^+^) (**d**) and effector T-cells (T_EFF_; CD127^-^KLRG1^+^) (**e**) were determined. Time points represent mean values with +SEM; all groups per time point *n* = 5. Data were analysed by two-way ANOVA followed by Dunnett´s multiple comparison test. Statistically significant differences were indicated among naive and vaccinated groups; (o: statistically significant worse than naive; +: statistically significant better than naive).

**Fig. 10:**
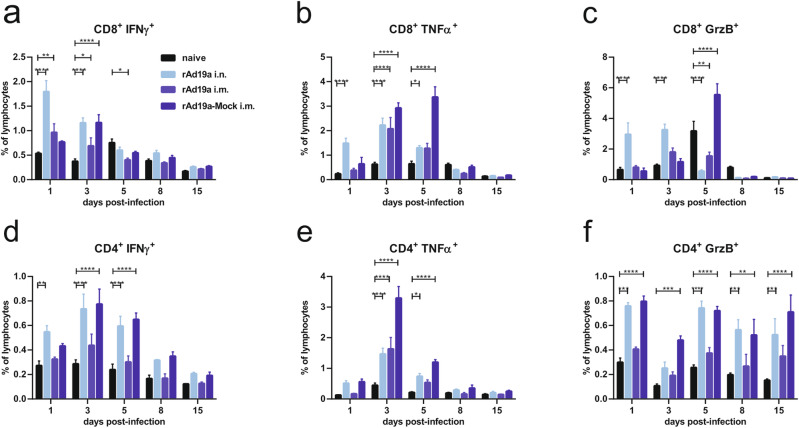
CD8^+^ and CD4^+^ T-cell response in the lung during early RSV infection in dependency of prior vaccination. Balb/c mice were immunized and challenged as described in Fig. 6 and lymphocytes from lungs were examined without restimulation on respective days post-infection. CD8^+^ and CD4^+^ T-cell responses were analysed by intracellular staining for IFNγ (**a** and **d**), TNFα (**b** and **e**), and granzyme B (**c** and **f**). Percentages of lymphocytes of different populations among CD8^+^ and CD4^+^ T-cells are shown (gating seen in Supplementary Fig. 6). Bars represent mean values with +SEM; all groups per time point *n* = 5. Data were analysed by one-way ANOVA followed by Tukey´s multiple comparison test. Statistically significant differences were indicated among all groups (*p < 0.05; **p < 0.005; ***p < 0.0005; ****p < 0.0001).

In more detail, we followed the anamnestic F-specific T-cell response in the lung by pentamer staining and intracellular cytokine staining. As expected, F-specific CD8^+^ T-cells were hardly detectable in naive animals over the course of infection. In rAd19a-Mock-treated animals, there was a steady increase of F-specific CD8^+^ T-cells until the end of the observation period (day 15). Interestingly, the absolute numbers of F-specific CD8^+^ T-cells in the lung and the dynamics were comparable for the two protected groups, which received the rAd19a-F boost either intranasally or intramuscularly over time. The response peaked at around day eight post-infection (Fig. 9a). However, a closer look on the phenotype of those cells revealed significant differences between the groups. As shown previously, solely the intranasal boost with rAd19a-F induced substantial numbers of F-specific T_RM_ that were clearly detectable at day one post-infection and then increased by a factor of 10 in numbers until day eight post-infection. At each of the time points, T_RM_ were the most prevalent phenotype in this group (Fig. 9b). In contrast, the anamnestic CD8^+^ T-cell response in the rAd19a-F i.m. cohort was characterized by an early expansion of effector T-cells, effector memory (T_EM_) and T_CM_, whereas local T_RM_ became detectable not earlier than day five post-infection. For all subpopulations, there was a decline in absolute numbers from day eight to day 15 in both protected groups. Interestingly, this was also true for the rAd19a-Mock treated animals with the exception of a steady increase in T_RM_ until day 15 (Fig. 9b–e). The rapid anamnestic response by T_RM_ induced by the intranasal rAd19a-F boost was mirrored in the kinetic of the cytokine-producing F-specific T-cells. Already at day one post-infection, high frequencies of IFNγ-, TNFα- and granzyme B (GrzB)-producing CD8^+^ T-cells were detectable reaching the maximum at day one or three post-infection before declining (Fig. 10a–c). In contrast, the mock group had the highest frequencies of TNFα^+^ and GrzB^+^ CD8^+^ T-cells at day five post-infection, which also marked the peak of protein content in the BAL of these animals as indirect measure of tissue damage (Fig. 10b, c). Interestingly, no significant differences in the frequencies of F-reactive CD8^+^ T-cells were found between the immunized and non-immunized animals at day eight and day 15.

At the level of F-specific CD4^+^ T-cells, the frequencies of IFNγ- and GrzB-producing cells were comparable between the rAd19a-F intranasally treated animals and the rAd19a-Mock treated ones over the whole observation period, whereas there were less reactive cells in the intramuscularly boosted animals (Fig. 10d, f). In the mock treated animals, the highest frequencies of TNFα-producing CD4^+^ T-cells were detected at day three post-infection preceding the CD8^+^ T-cell response by two days (Fig. 10e, b). Similar to the CD8^+^ T-cell response, rapid IFNγ production by vaccine-induced CD4^+^ T-cells (day one) was only detectable in animals that received the rAd19a-F boost intranasally, potentially indicating the presence of CD4^+^ T_RM_ in the lung (Fig. 10d).

**Fig. 11:**
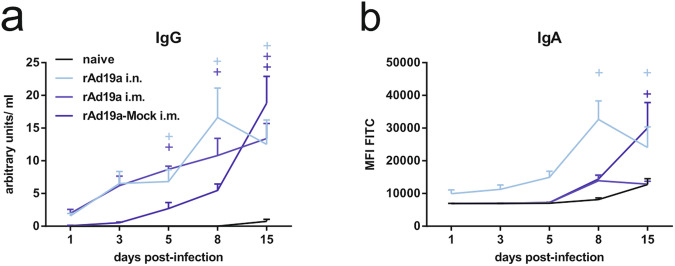
Humoral immune response during early RSV infection in dependency of prior vaccination. Balb/c mice were immunized and challenged as described in Fig. 6. F-specific IgG (**a**) and IgA (**b**) were examined in BALF by a flow cytometric assay using a 293A cell line stably expressing F. Time points represent mean values with +SEM; all groups per time point *n* = 5. Data were analysed by two-way ANOVA followed by Dunnett´s multiple comparison test. Statistically significant differences were indicated among naive and vaccinated groups; (o: statistically significant worse than naive; +: statistically significant better than naive).

Finally, we monitored local antibody dynamics in the challenged animals alongside the cellular response (Fig. 11, Supplementary Fig. 5). Both groups with the rAd19a-F boost showed high amounts of F-specific IgG in the BALF as early as day one post-infection, whereas the IgG levels in the mock group were substantially lower. In all immunized groups, an anamnestic response was visible by a 5-30-fold increase of F-specific IgG (Fig. 11a). There was no evidence for preferential boosting of either preF- or postF-specific antibodies after the challenge in the vaccinated groups. Interestingly, in sharp contrast, the primary RSV infection in naïve animals induced mainly antibodies binding to the F protein in post-fusion conformation and rarely antibodies recognizing the preF protein (Supplementary Fig. 5).

Similar to the presence of vaccine-induced T_RM_, F-specific IgA antibodies were only detected at early time points in animals having received the mucosal booster immunization. In these animals, the IgA levels also reached their maximum at day eight and then declined until day 15. In contrast, IgA antibody levels increased in the mock group over the whole observation period, which again mirrored the kinetic of the local CD8^+^ T_RM_ compartment. The rAd19a-F intermuscular treated animals showed only a marginal and temporary increase in IgA antibodies starting at day eight (Fig. 11b). In line with the IgG response, IgA antibodies in all vaccinated groups were able to bind pre- and post-fusion F, whereas the primary IgA response in naïve animals is dominated by postF-specific antibodies (Supplementary Fig. 5).

## Discussion

Recently, two new prophylactic RSV vaccines were FDA-approved for the prevention of severe RSV infection. However, effective mucosal vaccines against respiratory viruses are still missing to potentially reduce virus transmission^45^. Since several recombinant adenoviruses show a tropism for cells of the respiratory tract, replication-defective adenoviral vectors have been suggested to be promising candidates for intranasal or oropharyngeal immunizations^42,57–59^. Here we evaluated a novel vector platform based on the rare human serotype rAd19a as a heterologous booster immunization. Initially, we compared the immunogenicity and efficacy of RSV-F encoding rAd19a vectors with those of rAd5-based vaccine vectors, which have been proven to be highly immunogenic, but demonstrated some drawbacks in early clinical vaccine trials^51–53^.

We could show that rAd19a can induce humoral and cellular responses. While booster immunizations with rAd5-based vectors resulted in slightly stronger mucosal CD8^+^ T-cell responses, rAd19a-based vectors were more efficient in inducing circulating CD8^+^ T-cells in combination with consistently superior CD4^+^ T-cell responses in both compartments. This was in line with our previous findings on rAd19a-based vector vaccines against Influenza A Viruses^50^. Importantly, antibodies induced by both vectors were capable to bind both conformation of the F protein, preF and postF, which is in line with previous reports on serological responses to mRNA vaccines encoding the WT RSV F sequence^60^.

The differential profiles in the antibody and the T-cell responses might be due to the different tropisms of the two adenoviral vectors. Adenovirus vector 5 belongs to the subgroup C and binds to the coxsackie-and-adenovirus receptor (CAR) to enter a host cell^61,62^. This receptor is expressed on different cell types like epithelial cells^63^, but not on hematopoietic cells^64^. In contrast, sialic acids act as entry receptor for the subgroup D adenovirus vector 19a^65^ A heavily sialylated mucus layer in the respiratory tract is a barrier for sialic acid-binding pathogens. This might impact on viral entry^66^ and consequently on the antigen amounts in the lung, which may explain the lower number of CD8^+^ T_RM_ after rAd19a intranasal boost compared to rAd5. However, the increased number of polyfunctional CD8^+^ and CD4^+^ T-cells in the spleen and the higher amount of polyfunctional CD4^+^ T-cells in the lung suggest an efficient antigen presentation by DCs in the lymph nodes. Since DCs carry large numbers of sialylated glycans on their surface as potential interaction partners for the Ad19a fibre protein^67^, this could result in more efficient transduction of DCs by rAd19a compared to Ad5, as it has been shown for human monocyte-derived DCs^68^. Furthermore, rAd19a may be more immunogenic in humans than in mice. CD46 functions as an additional cellular attachment receptor for the subgroup D adenoviruses, which is present on all human cells^69^ but is absent in mice except their testis^70^. CD46 may be a potential entry receptor for rAd19a-based vector vaccines, which makes them even more promising for the use in humans.

Independent of the chosen vector, a mucosal boost vaccination provides efficient protection against RSV-induced pathogenesis in our challenge experiments. Interestingly, the outcome of the intramuscular immunization was slightly different for the two adenoviral vectors. The intramuscular immunization with rAd5-F were comparable effective in controlling viral replication and preventing RSV-induced weight loss than the two intranasally applied vectors. In contrast, animals that received an intramuscular rAd19a-F boost showed increased weight loss upon RSV infection even compared to naïve mice within the first five days post challenge. While the presence of local T-cells and IgA responses might explain the superior protection seen in intranasally immunized animals, the higher levels of systemic antibodies and T-cells after intramuscular Ad5 vaccination potentially lead to more efficient restriction of viral replication. Although not formally shown by viral load measurements, the significant higher increase of anti-F antibodies in the rAd19 i.m. immunized animals indirectly supports the presence of higher antigen loads during the infection. Furthermore, the significant higher levels of F-specific IgG2a antibodies after the vaccination with rAd5-F might indicate superior viral control via Fc-mediated effector functions, such as ADCC or ADCP, which depends very much on the IgG subclass and its FcγR binding properties^71^. However, since the adenoviral vector platform of Ad5 was already extensively studied in numerous studies, we focused on the differential outcome of the rAd19a booster immunization depending on the route of application. Interestingly, all rAd19a-immunized mice showed significant higher weight loss than previously naïve animals during the initial phase of the infection (days 2-3), but then intranasally immunized animals gained weight rapidly as also the viral titers decline. In contrast, in naïve animals steady weight loss and onset of reduced oxygen saturation was first observed between day 4 and 5 post infection, which indicated also the peak of viral replication. Since the initial weight loss does not directly correlate with viral loads and 0_2_ saturation, it might be also a consequence of the high energy needs during the anamnestic immune response in vaccinated mice and an adaption of metabolic processes, similar to infection-mediated cachexia as described for LCMV^72^. Unfortunately, our study could not finally address the exact mechanism for the weight loss and more detailed follow-up studies needed to be performed. However, we provided substantial evidence for immune-mediated mechanisms as major driver of the observed enhanced disease phenotype. Our detailed kinetic study revealed an early activation of RSV-specific lung T_RM_, induced by the mucosal boost immunization, which probably initiated an antiviral environment and a fast inhibition of virus replication^73^. This is underlined by an early presence of CCL5, CXCL9, IFNγ as well as IFNγ- producing T-cells in the respiratory tract one day post RSV infection. A systemic booster immunization with rAd19a-F did not result in such an early response. In contrast, higher levels of pro-inflammatory factors like IL-6, GM-CSF and CXCL1 were observed in BALF along with lower amounts of IFNγ and IFNγ-producing T-cells one day post-infection. However, this group displayed a pronounced infiltration of circulatory T-cells into the lung at later stages of the infection. Therefore, a lack of early immune correlates like T_RM_ and IgA, together with an excessive infiltration of T_CM_ at later time points may have provoked a vaccine-enhanced RSV disease in the intramuscularly boosted group. This ERD phenomena observed during the acute infection was even more pronounced in the group which received the F-specific DNA prime and a rAd19a-Mock boost (rAd19a-Mock encoding for IAV NP). Here, we suspect two mechanisms that contribute to that. First, a prime-only immunity is significantly less efficient in clearing the RSV infection leading to higher viral loads, which then potentiates the re-call response. Second, the prime-only immunity is initially weak but able to expand upon RSV infection, resulting in a pronounced T-cell infiltration with relatively high numbers of TNFα- and GrzB-producing T-cells. This is in line with other studies having shown that vaccine-induced circulating TNFα-producing CD4^+^ and CD8^+^ T-cells mediate weight loss, pulmonary dysfunction and airway obstruction upon RSV infection^27,74,75^. Additionally, high levels of GrzB-producing CD8^+^ T-cells potentially contribute to enhanced tissue damage^76–78^. These effects might cumulate to the observed disease amplitude and could be prevented by a pre-existing mucosal T-cell response.

Interestingly, while the T_RM_ and local IgG and IgA responses peaked around day eight after a rAd19a-F boost and then declined until day 15, a steady increase was observed in the rAd19a-Mock immunized animals. At the end of the observation period, IgA and CD8^+^ T_RM_ in the mock group exceeded the values of the rAd19a-F intramuscular group. The higher degree of viral replication and local antigen presence seemed to induce a very prominent secondary local response, which was not the case after an intramuscular rAd19-F boost. In the mock group, all other CD8^+^ memory populations were refractory at day 15. This potentially indicates some coordinated processes in the development of T_RM_ and local plasma cells^79^.

Moreover, we observed a local pre-existence and subsequently a rapid increase of IgA upon RSV infection in intranasally boosted animals. IgA serves as a first line of pathogen defense in the respiratory tract^47,80–84^, and is able to slow down an early infection^85^. This might be an additional reason for the rapid viral control in this group. Since the IgG responses were comparable in regard to the quantity and the IgG subclass distribution after intranasal and intramuscular boost immunization, IgG-mediated effector functions might be less likely responsible for the differential degree of disease control. Furthermore, the anamnestic response to preF and postF proteins were comparable. However, we have not formally excluded differences in regard to antibody avidity or Fc-mediated effects in functional assay, which could have also impacted on the disease progression.

Interestingly, the primary antibody response induced by the RSV infection in non-vaccinated animals is preferentially directed against the postF protein. This might be relevant for secondary RSV infection and could potentially explain why vaccine-induced immune responses can provide superior protection compared to natural RSV immunity^37^. Even in the Ad19a-Mock immunized, the RSV-F specific IgG and IgA antibodies detected after the infection are capable to bind preF and postF proteins, underlining the characteristics of an anamnestic antibody response initially induced by the RSV-F encoding DNA vaccine.

Taken together, we introduced rAd19a as a promising alternative to rAd5 in mucosal vaccinations. Although it is slightly less immunogenic in mice, this might be different in humans due to the broad expression of the CD46 receptor on hematopoietic cells^69,70^. The use of rAd5 as an immunization vector raises several challenges in the human population. Its high seroprevalence of 60–90%^51^ might result in neutralization of the vaccine vector upon immunization and therefore a dampened immunogenicity^51,86,87^. In contrast, Ad19a is a rare adenovirus subtype, with a seroprevalence of about 17%^88,89^. Its use as viral vaccine vector may circumvent preexisting anti-vector immunity^90^.

Additionally, we could prove that a systemic DNA-prime followed by mucosal adenoviral boost vaccination provides an efficient immunity against RSV infection in terms of virus replication and disease symptoms. This goes in hand with the IAV data by Lapuente et al.^50^ and SARS-CoV-2 data by Freitag et al.^91^. They could show that an intramuscular prime followed by an intranasal boost was more efficient in inducing a robust immune response compared to a single mucosal vaccine application.

During the pandemic, the intramuscular administration of the adenovirus-based vaccines ChAdOx1 nCoV-19 (AstraZeneca) and Ad26.COV2.S (Janssen) raised some safety concerns, because vaccine-induced thrombotic thrombocytopenia (VITT) appeared as a rare, but severe side effect^92,93^. Circulating adenoviral vectors and their components may interact with platelets and cause platelet activation and the release of platelet factor 4 (PF4). These results in an autoimmune reaction with high-titer IgG directed against PF4 and intense platelet activation and thrombin generation^93,94^. In a pre-clinical study Freitag et al. could detect adenoviral DNA in the liver and spleen of mice after an intramuscular immunization with an Ad5-based vaccine. Interestingly, their sensitive PCR assay could not show any indication of a systemic DNA spread after an intranasal Ad5 application of the same vaccine dose^91^. Microvascular damage after intramuscular vaccine injection benefits VITT^95^, and is less likely by an intranasal route. This leads to the assumption, that an intranasal vaccine delivery reduces the risk for the development of VITT compared to an intramuscular one.

With these conclusions, we would like to further encourage the development of mucosal vaccine strategies against existing and newly emerging respiratory viruses. Such vaccines might be especially important for the development of an urgently needed RSV prophylaxis, since mucosal immunity induces highly protective immune responses while limiting mechanisms of ERD.

## Methods

### Plasmids and adenoviral vectors

The plasmid encoding the codon-optimized sequence of the full-length WT RSV F protein (GenBank database entry EF566942), referred to as pV-Fsyn, has been described previously^96^. DNA vaccines for immunization were prepared using the PureLinkTMHiPurePlasmidMaxiprep Kit (Invitrogen by Thermo Fisher Scientific) according to the manufacturer’s instructions. As modification, two additional steps were added to remove residual endotoxin. After clarifying the bacterial lysate, the DNA-containing solution was incubated with 3 ml of endotoxin removal buffer A (50 mM MOPS (pH 7.0), 750 mM NaCl, 10% (w/v) triton-x 100, 20% (v/v) isopropanol) for 15 min on ice. Afterwards the DNA was bound on the Hi pure columns and washed with 30 ml endotoxin removal buffer B (100 mM C_2_H_3_NaO_2_ (pH 5.0), 750 nM NaCl, 1% (w/v) triton x 100) before continuing with the manufacturer´s protocol.

The replication-deficient (ΔE1ΔE3) recombinant adenoviral vectors based on the serotypes 5 (Ad5(Pro)-CMV-RSV-F) or 19a (Ad19a(Pro)-CMV-RSV-F), encoding the same codon-optimized RSV-F sequence, were provided by Sirion Biotech GmbH (Martinsried, Germany). As control vectors, Ad5-NP (Ad5-CMV-NP) and Ad19-NP (Ad19a-CMV-NP) encoding nucleoprotein (NP) from the IAV strain H1N1 A/Puerto Rico/8/34 were used^50^.

To ensure comparable expression levels of RSV-F from both adenoviral vectors, non-complementary, A549 cells (human lung epithelial cell line) were transduced with rAd5-F and rAd19a-F, respectively, with an MOI of 10. Two days later, cell lysates were prepared and Western Blot analyses were performed to detect RSV-F under non-reducing conditions as described before^97^. Additionally, intact cells were stained with Monoclonal Anti-RSV-Pre-F0 specific Antibody (2 µg/ml, 4 °C, 30 min, RS0-Y132, acrobiosystems) followed by anti-mouse IgG1-APC (1:300, 4°, 30 min, RMG1-1, Biolegend) to confirm the presence of pre-F on the surface of the transduced cells by flow cytometric analyses.

### Mice and immunization

6-8 weeks old female BALB/cJRj mice were purchased from Janvier (Le Genest-Saint-Isle, France) and housed in individually ventilated cages according to the national law and institutional guidelines. The study was approved by external ethics committees authorized by the Government of Lower Franconia (license 55.2-2532-2-906) or by the North Rhine-Westphalia State Office for Consumer Protection and Food Safety (license 84–02.04.2013-A371). The research staff was trained in animal care and handling in accordance to the FELASA and GV-SOLAS guidelines.

The intramuscular (i.m.) immunization in mice was performed under light anaesthesia with inhaled isoflurane. In case of DNA immunizations, 10 µg of plasmid DNA was diluted in 60 µl PBS and a volume of 30 µl was injected in the gastrocnemius of each hind leg, followed by electroporation as described elsewhere^98^. 28 days later, mice were boosted either intramuscularly (in 60 µl PBS) or intranasally (in 50 µl PBS) with a dose of 2 ×10^6^ IU of the adenoviral vectors. For intranasal (i.n.) immunization, the vaccines were slowly pipetted into one nostril under general anaesthesia (100 mg/kg ketamine and 15 mg/kg xylazine). In all experiments, unvaccinated animals (naive) serve as control to define background levels in immunological assays or the course of infection in non-treated animals.

Blood samples were collected from the retro-orbital sinus using glass capillaries. For final analysis, mice were euthanized by inhaled isoflurane. The tracheae were cannulated and BALF were collected by rinsing the lungs twice with 1 ml PBS. Afterwards, lungs and spleens were collected.

### FACS-based antibody analysis

A stably-transduced, inducible 293A cell line, which expresses full-length, transmembrane RSV-F after the addition of doxycycline (400 ng/ml for 24 h) was used to detect F-specific antibodies as previously described^37^. Briefly, 10^5^ cells were incubated with sera or BALF diluted in FACS-PBS (PBS with 0.5% BSA and 1 mM sodium azide) and bound RSV-F specific antibodies were detected using polyclonal anti-mouse IgG-FITC (1:300, 4 °C, 20 min; Poly4060, BioLegend) or anti-mouse IgA-FITC (1:300, 4 °C, 20 min; C10-3, BD Bioscience). For the quantification of F-specific IgG1 and IgG2a antibodies, IgG subclass-specific secondary antibodies were used, namely anti-mouse IgG1-APC (1:300; RMG1-1, BioLegend) and anti-mouse IgG2a-PerCP (1:300; RMG2a-62, BioLegend). Samples were measured on an AttuneNxt flow cytometer (ThermoFisher) and analysed using FlowJoTM software (Tree Star Inc.). To determine the concentration of F-specific IgGs, serial dilutions of a reference mouse serum (IgG: 355 arbitrary units/µl; IgG1: 202 µg/ml; IgG2a: 28 µg/ml) were run as standard in each measurement.

### ELISA for the detection of preF and postF-binding antibodies

96-well ELISA plates (Lumitrac, high binding, Greiner Bio-One) were coated with 100 ng/well HRSV(A) Pre-fusion glycoprotein (acrobiosystems; #RSF-V52H7) or HRSV(A) Post-fusion glycoprotein (acrobiosystems; #RSF-V52H6) diluted in carbonate buffer overnight at 4 °C. Afterwards, free binding sites were blocked with 5% skimmed milk in PBS-T_0.05_ (PBS containing 0.05% Tween-20, Sigma-Aldrich). After a washing step with PBS-T_0.05_, diluted sera or BAL were added and incubated for one hour at room temperature. Subsequently, plates were washed and the detection antibodies, HRP-coupled polyclonal anti-mouse IgG (1:3000, PA1-84631, Invitrogen) or anti-mouse IgA (1:5000, A90-103P, Bethyl Laboratories), were added for one hour. After washing with PBS-T_0.05_ and the addition of an ECL substrate, the signals were acquired on a microplate luminometer (VICTOR X5, Perkin Elmer) with the PerkinElmer 2030 Manager software.

### RSV microneutralization assay

To determine RSV-specific neutralizing antibody titers, 2-fold serial dilutions of complement-inactivated mouse sera (56 °C, 30 min) were pre-incubated at 37 °C for 1 h with 200 PFU of a GFP-expressing RSV reporter^99^. In the next step, 100 µl of serum-virus mix was applied to A549 cells, which had been seeded in 100 µl DMEM containing 1% FCS, 1% penicillin-streptomycin and 1% GlutaMaxTM-I(100x) (gibco) at 1 ×10^4^ cells/well in a flat-bottom 96-well plate the day before. After 72 h, infected cells expressing GFP were counted by an ImmunSpot® Fluorescent Analyzer (Cellular Technology Limited). 50% plaque reduction neutralization titres (PRNT_50_) were defined as the reciprocal value of the highest serum dilution that inhibited more than 50% of plaques observed in infected control wells without serum/BALF treatment on the same plate.

### Tissue preparation

For final analyses, spleens and lungs were harvested. The latter were cut into small pieces and digested for 45 min at 37 °C with 500 units Collagenase D and 160 units DNase I in 2 ml R10 medium (RPMI 1640 supplemented with 10% FCS, 2 mM L-Glutamine, 10 mM HEPES, 50 µM β-mercaptoethanol and 1% penicillin/streptomycin). Lungs and spleens were mashed through a 70 µm cell strainer. Erythrocytes were lysed by incubation in ammonium-chloride-potassium (ACK solution, Lonza) for 8 min. After washing, the cells were resuspended in R10 medium, counted and used for further analyses. Regularly, one million splenocytes or 20% of the lung cell suspension were used for the T-cell assays described in the following.

### Intracellular cytokine staining

Six weeks after the boost immunization, animals were sacrificed to analyse lymphocytes from lung and spleen tissue as described elsewhere^41^. Briefly, cells were re-stimulated for 6 h in the presence of monensin (2 µM), anti-CD28 (1 µg/ml; 37.51, eBioscience), anti-CD107a-FITC (1:200; 1D4B, BD Bioscience) and 5 µg/ml of each RSV-F peptide mix. The CD4 mix consisted of F_48-62_ (LRTGWYTSVITIELS), F_52-66_ (WYTSVITIELSNIKE), F_183-197_ (NGVSVLTSKVLDLKN), F_187-207_ (VLTSKVLDLKNYIDK), and the CD8 mix of F_80-94_ (KQELDKYKNAVTELQ), F_84-98_ (DKYKNAVTELQLLMQ), F_243-257_ (VTTPVSTYMLTNSEL), F_247-261_ (VSTYMLTNSELLSLI). Non-stimulated lymphocytes were used as controls for unspecific cytokine production and background subtraction. After stimulation, the surface staining was performed with anti-CD8a-Pacific blue (1:2000; 53-6.7, BD Biosciences), anti-CD4-PerCP (1:2000; RM4-5, Thermofisher), and Fixable Viablilty Dye eFluor® 780 (1:4000; eBioscience) in FACS-PBS for 20 min at 4 °C. After fixation (2% paraformaldehyde, 20 min, 4 °C) and permeabilization (0.5% saponin in FACS-PBS, 10 min, 4 °C), cells were stained intracellularly with anti-IL-2-APC (1:300; JES6-5H4), anti-TNFα-PECy7 (1:300; MP6-XT22), and anti-IFNγ-PE (1:300; XMG1.2, each BioLegend).

For ex vivo analysis, intracellular cytokine staining was done without in vitro re-stimulation. The surface staining was performed with anti-CD4-BV510 (1:100; RM4-5, BD Biosceince), anti-CD8a-BV605 (1:500; 53-6.7, BioLegend), anti-CD107a-PerCP (1:200; 1D4B, eBioscience), anti-CD49b-PE (1:300; DX5, eBioscience), anti-CD45.2-PE/Dazzle (1:500; 104, Biolegend), anti-CD19-PE-Cy7 (1:300; 1D3, BD Bioscience), and Fixable Viablilty Dye eFluor® 780. After fixation and permeabilization, intracellular staining was done using anti-Granzyme B-eFlour® 450 (1:500; NGZB, eBioscience), anti-TNFα-AF488 (1:300; MP6-XT22, BD Bioscience), and anti-IFNγ-BV711 (1:200; XMG1.2, BioLegend). Data were acquired on an AttuneNxt flow cytometer (ThermoFisher) and analysed using FlowJoTM software (Tree Star Inc.). The gating were performed as described recently^100^.

### Intravascular staining and T-cell phenotype analyses

Intravascular staining was performed to discriminate between circulating and tissue-resident memory T-cells, as described by Anderson et al.^101^. Briefly, 2 µg anti-CD45-BV510 (30-F11, BioLegend) diluted in 150 µl PBS were injected into the tail vein three minutes before euthanizing mice with inhaled isoflurane. TRM are identified by the absence of anti-CD45-BV510 labelling.

Lymphocytes were isolated from lung tissue as described above. One fifth of the cell suspension was incubated with APC-labelled H-2Kd F_85-93_ pentamer (1:40, ProImmune) for 20 min at 4 °C in FACS-PBS, followed by a second surface staining step with anti-CD127-FITC (1:500; A7R34, BioLegend), anti-CD103-PE (1:200; 2E7, Invitrogen), anti-CD69-PerCP-cy5.5 (1:300; H1.2F3, BD Bioscience), anti-KLRG1-PE-Cy7 (1:300; 2F1, eBioscience), and anti-CD8a-BV711 (1:300; 53-6.7, BioLegend). The gating were performed as described recently^100^.

For the phenotypic T-cell analyses during the RSV challenge, the intravascular staining was omitted and the following panel was used for staining: anti-CD45-BV510 (1:500; 30-F11, BioLegend), anti-CD103-BV605 (1:100; 2E7, BioLegend), anti-CD8a-BV702 (1:200; 53-6.7, BioLegend), anti-CD127-FITC, anti-CD69-PerCP-Cy5.5 (1:200; H1.2F3, BioLegend), anti-CD44-PE (1:5000; IM7, BD Bioscience), anti-KLRG1-PE-Cy7 (1:300, 2F1, eBioscience) and Fixable Viablilty Dye eFluor® 780. Data were acquired on an AttuneNxt flow cytometer (ThermoFisher) and analysed using FlowJoTM software (Tree Star Inc.).

### RSV-A2 infection

Six weeks after the boost immunization mice were anesthetized (100 mg/kg ketamine and 15 mg/kg xylazine) and challenged intranasally by slowly pipetting RSV-A2 (5 ×10^6^ PFU) diluted in 50 µl PBS into one nostril. The weight loss was monitored daily after infection as an indicator of disease progression. Blood oxygen saturation levels were measured with the MouseOxTM Pulse-oximeter (Starr Life Science). A minimum of 25 individual readings were taken with the throat-clip. BALF and lungs were collected for further analyses at the end of the experiment.

### Flow cytometric analyses of cellular infiltrate after RSV-A2 infection

A surface staining was done with anti-Ly6c-eFlour® 450 (1:300; KK1.4, Invitrogen), anti-CD11c-BV510 (1:100; HL3, BD Bioscience), anti-CD170-BV600 (1:300; 1RNM44N, Invitrogen), anti-CD103-BV711 (1:300; 2E7, BioLegend), anti-CD45-FITC (1:300; 30-F11, BioLegend), anti-MHC-II-PerCP-Cy5.5 (1:300; M5/114.15.2, BioLegend), anti-CD64-PE (1:300; X54-5/7.1, BioLegend), anti-Ly6G-PE-Cy7 (1:300; 1A8, BD Bioscience), anti-CD24-APC (1:300; M1/69, BD Bioscience), and anti-CD11b-APC-Cy7 (1:300; M1/70, BD Bioscience). Data were acquired on an AttuneNxt flow cytometer (ThermoFisher) and analysed using FlowJoTM software (Tree Star Inc.).

### RT-qPCR

Viral RNA was isolated from cell-free BALF using the NucleoSpin® RNA Virus Kit (Macherey-Nagel) according to the manufacturer´s instruction. The quantity of viral RNA copies was determined by reverse transcription quantitative PCR (RT-qPCR) using GoTaq® 1-Step RT-qPCR System Kit (Promega). The following primers were used to amplify a sequence from the RSV nucleoprotein (for: AGATCAACTTCTGTCATCCAGCAA; rev: GCACATCATAATTAGGAGTATCAAT). In vitro transcribed RNAs were used as standards and the detection limit was 10 copies per reaction, which corresponds to 667 copies/ml BALF.

### Statistical analysis

Results are shown as mean + standard error of the mean (SEM) or in log-scale figures as median ± interquartile range. Statistic comparisons were performed by unpaired two-tailed Student´s *t* test and one-way ANOVA test, followed by Tukey´s post test using Prism 6.0 (GraphPad Software, Inc.). A *p* value of <0.05 was considered to be statistically significant.

## Supplementary information

Supplementary information

## Data Availability

The datasets used and/or analysed during the current study available from the corresponding author on reasonable request.
