# Supplementary material for: Evaluation of adenoviral vector Ad19a encoding RSV-F as novel vaccine against respiratory syncytial virus
Source: NPJ Vaccines. 2024 Oct 29;9:205. doi: 10.1038/s41541-024-01001-z (PMC11522487; doi:10.1038/s41541-024-01001-z)
Supplement: Supplementary file 1 — Supplementary information [file 41541_2024_1001_MOESM1_ESM.pdf]

Supplementary Data

Suppl. Figure 1: Comparable expression levels after transduction with rAd5 to rAd19a

Suppl. Figure 2: PostF and PreF-reactive antibody responses after rAd5 and rAd19a boost

Suppl. Figure 3: IgG subclass analyses pre and post RSV challenge

Suppl. Figure 4: Comparison of ERD after boost with rAd5-Mock to rAd19a-Mock

Suppl. Figure 5: Kinetics of PostF and PreF-reactive antibody responses after RSV infection

Suppl. Figure 6: Gating schemes for flow cytometric analyses

Supplementary Figure 1

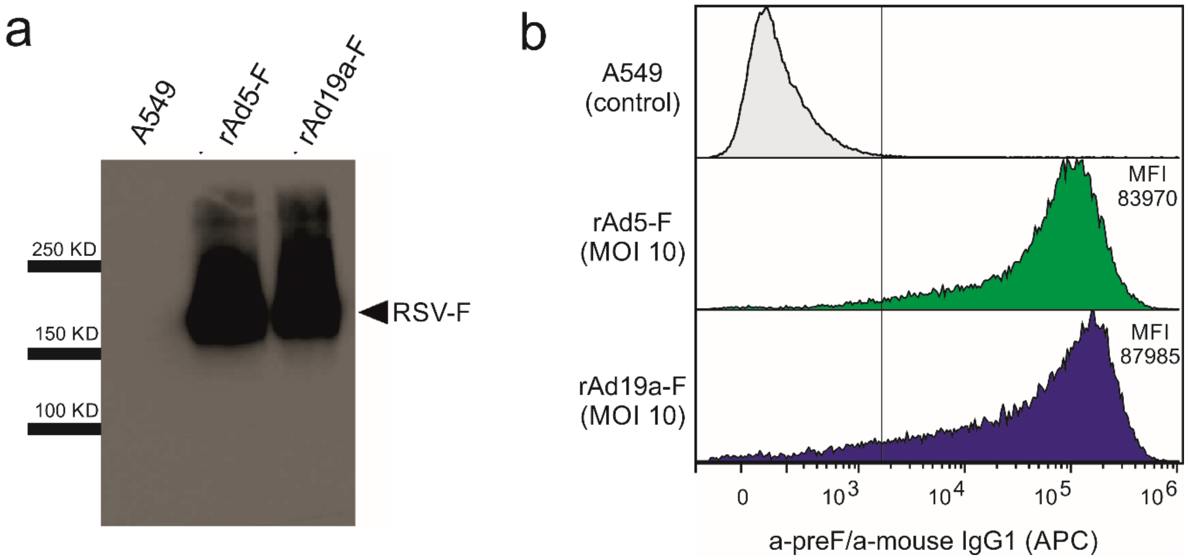

Suppl. Figure 1: Comparable expression levels after transduction with rAd5 to rAd19a

To confirm comparable expression levels after transduction with the two different adenoviral vectors, A549 cells were transduced with an MOI of rAd5-F or rAd19a-F respectively and two days later the cells were harvested. a) cell lysates were prepared and Western Blot analyses were performed under non-reducing conditions with monoclonal RSV-F antibody as described before (97). b) Additionally, intact cells were stained with monoclonal Anti-RSV-Pre-F0 specific Antibody followed by anti-mouse IgG1-APC to confirm the presence of pre-F on the surface of the transduced cells by flow cytometric analyses.

Supplementary Figure 2

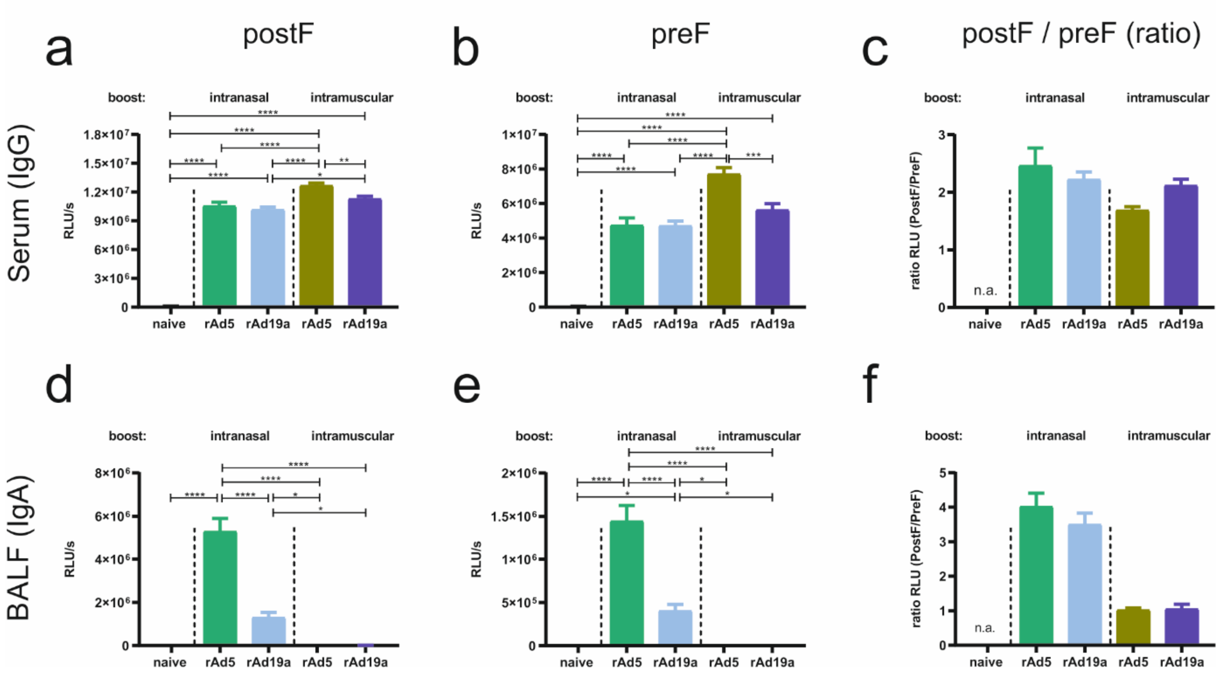

Suppl. Figure 2: PostF and PreF-reactive antibody responses after rAd5 and rAd19a boost.

BALB/c mice were primed intramuscularly with an F-encoding DNA plasmid (10 µg plasmid) followed by electroporation and boosted 28 days later either intramuscularly (i.m.) or intranasally (i.n.) with rAd5 or rAd19a viral vectors encoding for F (2 x 10^6 infectious units per vector). Serum antibody responses were analyzed 14 days and mucosal immune responses 36 days after boost immunization. PostF (a,d) and preF (b,e) -specific IgG (a-c) and IgA (d-f) were examined by ELISA using postF0 and preF0 proteins as coating reagents. Additionally the ratio of postF/preF were calculated by dividing the RLUs of the respective measurements (c, f). RLUs of naïve samples indicate the background values of the assay. Bars represent mean values with + SEM (Serum: rAd5 (i.n.) n = 11, rAd19 (i.n.) n = 12, other groups n = 18; BALF: rAd5 (i.n.) n = 11, other groups n = 12). Data were analyzed by one-way ANOVA followed by Tukey´s multiple comparison test. Statistically significant differences were indicated among all groups (*, p<0.05; **, p<0.005;***, p<0.001; ****, p<0.0001). n.a. = not applicable

Supplementary Figure 3

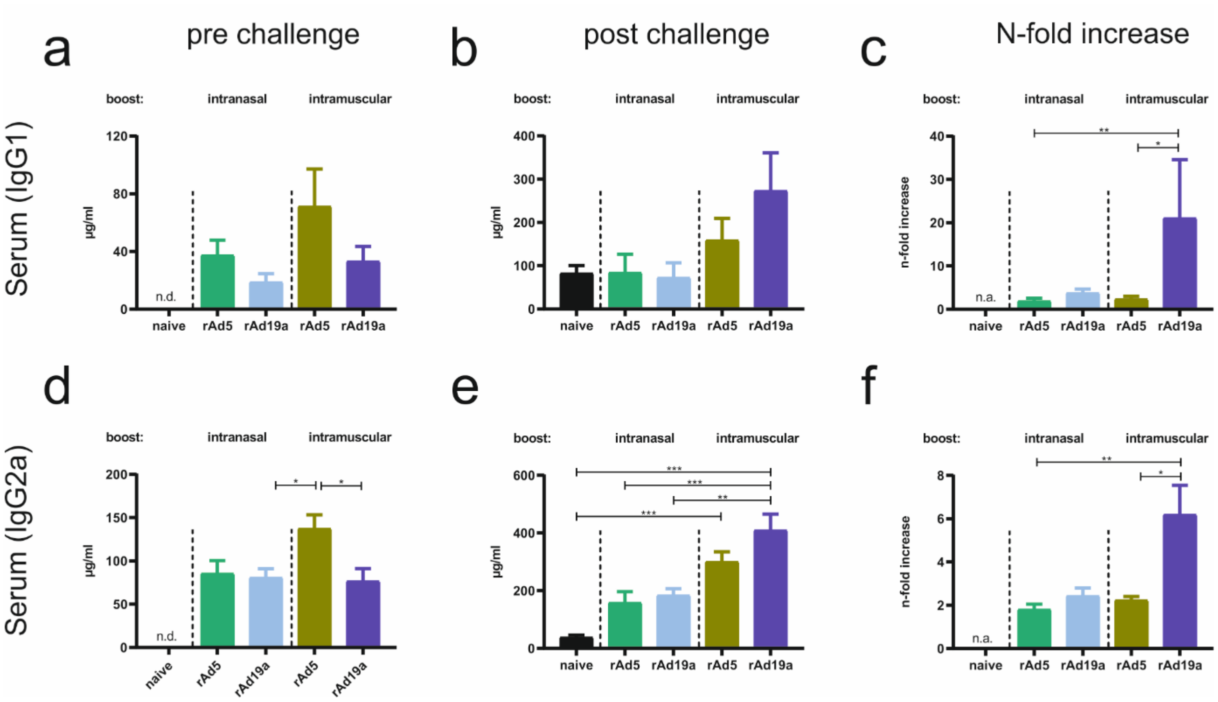

Suppl. Figure 3: IgG subclass analyses pre and post RSV challenge

Balb/c mice were immunized as described before. 37 days after boost immunization, mice were challenged with 5 x 10^6 PFU RSV-A. Serum were collected 14 days post immunization (pre challenge) and 21 days post challenge to determine the RSV-F specific IgG1 (a-c) and IgG2a (d-f) antibody response by a flow cytometric assay using a 293A cell line stably expressing F. The amount of IgG1 or IgG2 were calculated according to standard mouse serum together with the n-fold increase between pre and post challenge sera. Bars represent mean values with + SEM (n = 6). Data were analyzed by one-way ANOVA followed by Tukey´s multiple comparison test. Statistically significant differences were indicated among all groups (*, p<0.05; **, p<0.005; ***, p<0.001). n.d. = not detectable; n.a.= not applicable.

Supplementary Figure 4

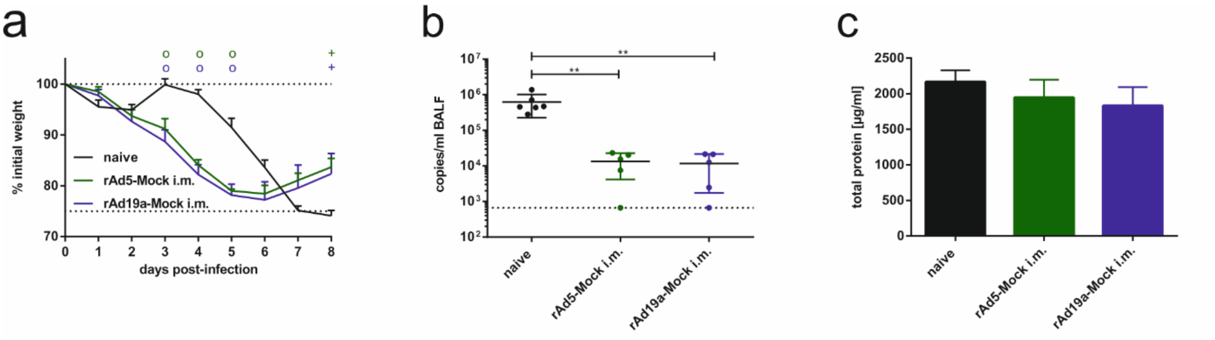

Suppl. Figure 4: Comparison of ERD after boost with rAd5-Mock to rAd19a-Mock

BALB/c mice were primed intramuscularly with an F-encoding DNA plasmid (10 µg plasmid) followed by electroporation and boosted 28 days later intramuscularly (i.m.) with rAd5 or rAd19a viral vector encoding for the influenza nucleoprotein NP (mock) (2×10^6 infectious units per vector). 37 days after mock immunization, all mice were challenged with 5×10^6 PFU RSV-A. (a) Animals were monitored daily for body weight. Time points show group´s mean values with +SEM; naive n = 6, other groups n = 5. Data were analyzed by Friedman test followed by Dunn´s multiple comparison test. Statistically significant differences were indicated among naive and vaccinated groups; (o: statistically significant worse than naive; +: statistically significant better than naive). (b) Tissue damage was indirectly measured by protein content in the BALF 8 days after infection. Bars represent mean values with ± SEM; naive n = 6, other groups n = 5. Data were analyzed by one-way ANOVA followed by Tukey´s multiple comparison test. (c) At day 8, viral loads in BALF samples were measured by qRT-PCR. Depicted are the individual copies/ml BALF with the group´s mean values with + SEM; naive n = 6, other groups n = 5. The qRT-PCR´s detection limit was 667 copies/ml BALF and is marked with a dotted line. Data were analyzed by one-way ANOVA followed by Tukey´s multiple comparison test. Statistically significant differences were indicated among all groups. (**, $p < 0.005$).

Supplementary Figure 5

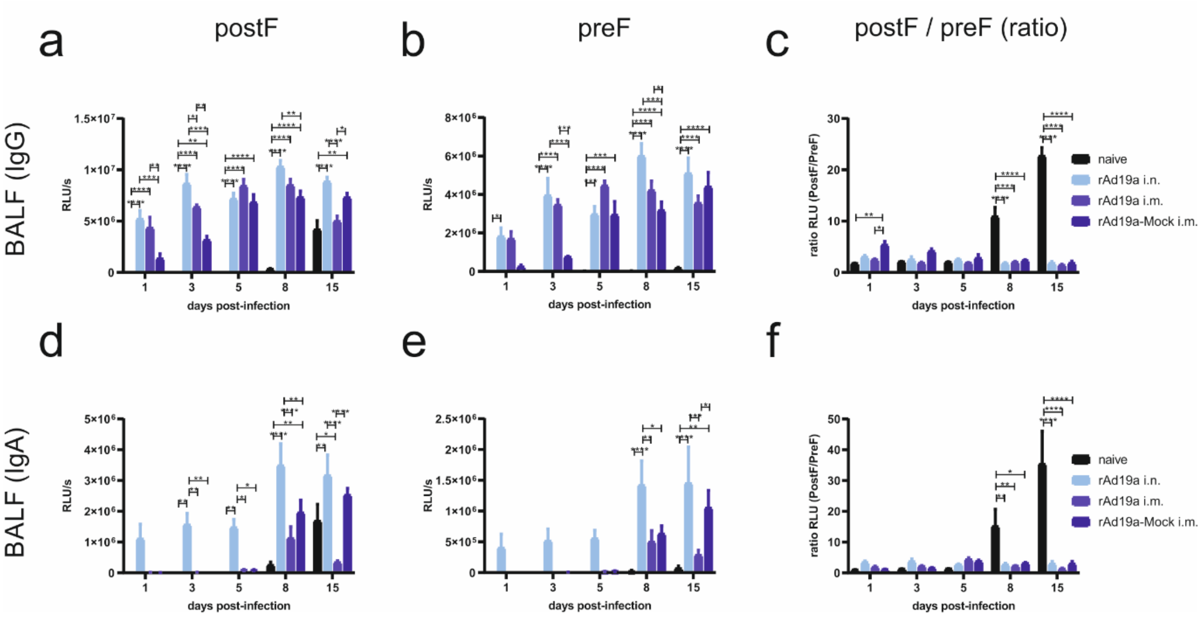

Suppl. Figure 5: Kinetics of PostF and PreF-reactive antibody responses after RSV infection

Balb/c mice were immunized and challenged as described in Figure 6. PostF (a, d) and PreF (b, e) specific IgG (a-c) and IgA (d-f) were examined in BALF by ELISA using postF0 and preF0 proteins as coating reagents. Additionally the ratio of postF/preF were calculated by dividing the RLUs of the respective measurements (c, f). Time points represent mean values with + SEM; all groups per time point n = 5. Data were analyzed by one-way ANOVA followed by Tukey´s multiple comparison test. Statistically significant differences were indicated among all groups (*, p<0.05; **, p<0.005; ***, p<0.001; ****, p<0.001).

Supplementary figure 6

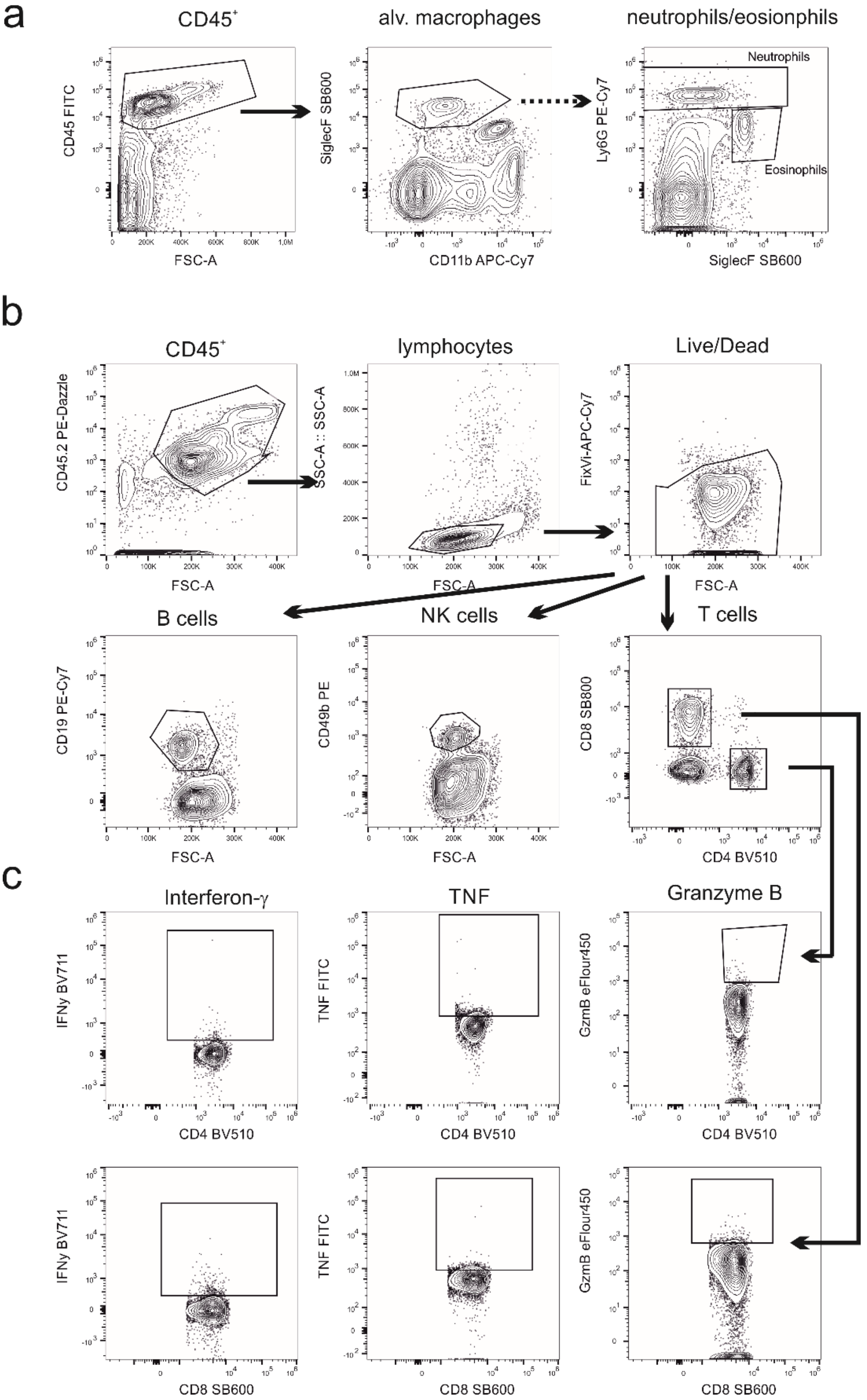

Suppl. Figure 6: Gating schemes for flow cytometric analyses

The contour plots with outliers shows representative examples for the gating schemes which have been used in this study to identify the neutrophils and eosinophils (a), the diverse lymphocytes populations (b) in Fig.8. Furthermore, representative stainings of the ex vivo T-cell analyses (c) presented in Fig. 9 are shown. Positive gating is indicated by solid arrows, negative gating (selected population is excluded from next gate) with dashed arrows.
